# A retrospective analysis of combined treatment with escitalopram and Naoan dropping pills treatment for depression

**DOI:** 10.1097/MD.0000000000042169

**Published:** 2025-04-25

**Authors:** Xiaoyan Wang, Yan Che, Wenli Wang

**Affiliations:** a Department of Ultrasound, The Third People’s Hospital of Tianshui, Gansu Tianshui, P. R. China.

**Keywords:** cerebral hemodynamics, depression, escitalopram, Naoan dropping pills, transcranial Doppler

## Abstract

This study evaluated the effectiveness of combining escitalopram with Naoan dropping pills for the treatment of depression, focusing on improvements in depressive symptoms, daily functional abilities, and cerebral hemodynamics. A total of 87 patients diagnosed with depression at The Third People’s Hospital of Tianshui over a 2-year period were retrospectively analyzed. They were divided into 2 groups according to whether Naoan dropping pills were used or not. Group A (n = 43) received only escitalopram, while group B (n = 44) received a combination of escitalopram and Naoan dropping pills for a period of 2 months. Efficacy was determined using the Hamilton depression scale (HAMD), with a ≥50% reduction in HAMD score from baseline considered effective. The activities of daily living (ADL) scale was employed to assess daily functional abilities. Cerebral hemodynamics were evaluated using transcranial Doppler ultrasound (TCD). By the end of the 2-month treatment period, Group B demonstrated a significantly higher efficacy rate (90.91%) than Group A (72.09%). In addition, Group B showed more pronounced improvements in ADL scores, indicating enhanced day-to-day functioning. TCD measurements further revealed higher systolic and diastolic blood flow velocities in the major cerebral arteries of Group B, suggesting improved cerebral perfusion. The combination of escitalopram and Naoan dropping pills proved more efficacious in ameliorating depressive symptoms, enhancing daily functional abilities, and improving cerebral hemodynamics than escitalopram alone. These outcomes highlight the potential benefits of integrated treatment strategies for the management of depression, advocating the adoption of personalized and comprehensive treatment modalities.

## 1. Introduction

Depression is one of the most prevalent clinical mental health conditions and is profoundly affected by a diverse array of factors, including genetics, intricacies in brain architecture, neurochemical dynamics, hormonal fluctuations, early life trauma, and the nuanced influence of cultural contexts.^[1]^ This condition manifests clinically as a persistent low mood, with severe instances potentially escalating to suicidal or homicidal behaviors, thus presenting a substantial threat to both societal well-being and the lives and health of those afflicted.^[2]^ A cross-sectional epidemiological study has shed light on the prevalence of depression in China, with an estimated prevalence of 6.8%. This study further revealed a sex disparity in the incidence of depression, noting that it is more prevalent among women than men.^[3]^ Moreover, a concerning observation from this study was the apparent inadequacy of treatment with only a fraction of patients receiving sufficient medical attention.^[3]^ These data highlight the critical need for enhanced awareness, improved access to mental health services, and the development of more effective treatment strategies to mitigate the impact of depression on individuals and society.

Escitalopram, a highly selective serotonin reuptake inhibitor (SSRI), has been validated to markedly enhances various cognitive functions in patients with depression, including verbal, nonverbal, and working memory functions.^[4,5]^ Its therapeutic benefits extend beyond cognitive improvement, effectively alleviating negative mood and elevating the overall quality of life of those afflicted.^[5]^ However, a notable proportion of patients do not respond adequately to SSRIs alone or experience residual symptoms, even at optimized dosages.^[6]^ Consequently, combination therapies or adjunctive treatments are being increasingly explored as potential strategies to augment treatment effects and improve patient outcomes. In recent years, growing attention has been directed toward integrating certain Traditional Chinese Medicine (TCM) formulations for the management of depression.^[7]^ Naoan dropping pills, a TCM product formulated from multiple herbal components, including chuanxiong (Ligusticum wallichii), safflower (Carthamus tinctorius), angelica (Angelica sinensis), ginseng (Panax ginseng), and borneol, have shown promise for promoting cerebral blood flow, protecting vascular endothelia, and exerting neuroprotective effects.^[8]^ Although their precise mechanisms are complex and likely multifaceted, these herbs have traditionally been used to enhance circulation, reduce blood stasis, and support qi balance, which may collectively influence both emotional regulation and brain physiology.

Growing evidence suggests that abnormalities in cerebral blood flow and impaired cerebral blood flow self-regulation (CBFSR) may contribute to the onset and perpetuation of depressive symptoms.^[1,9]^ Improved cerebral perfusion in specific brain regions (e.g., prefrontal cortex, hippocampus, and amygdala) has been associated with reduced depressive symptoms and better treatment outcomes.^[10,11]^ Consequently, exploring therapies that address both neurochemical imbalances and cerebral hemodynamics may yield novel insights into the treatment of depression.

Against this backdrop, this retrospective study aimed to evaluate the therapeutic potential of combining escitalopram with Naoan dropping pills to improve depressive symptoms and cerebral hemodynamics. The findings of this study may help illuminate the synergistic effects of combining a conventional antidepressant with a TCM formulation, potentially offering clinicians and researchers a broader spectrum of therapeutic possibilities for managing depression.

## 2. Methods

### 2.1. Participants and treatment protocols

**Figure 1.**
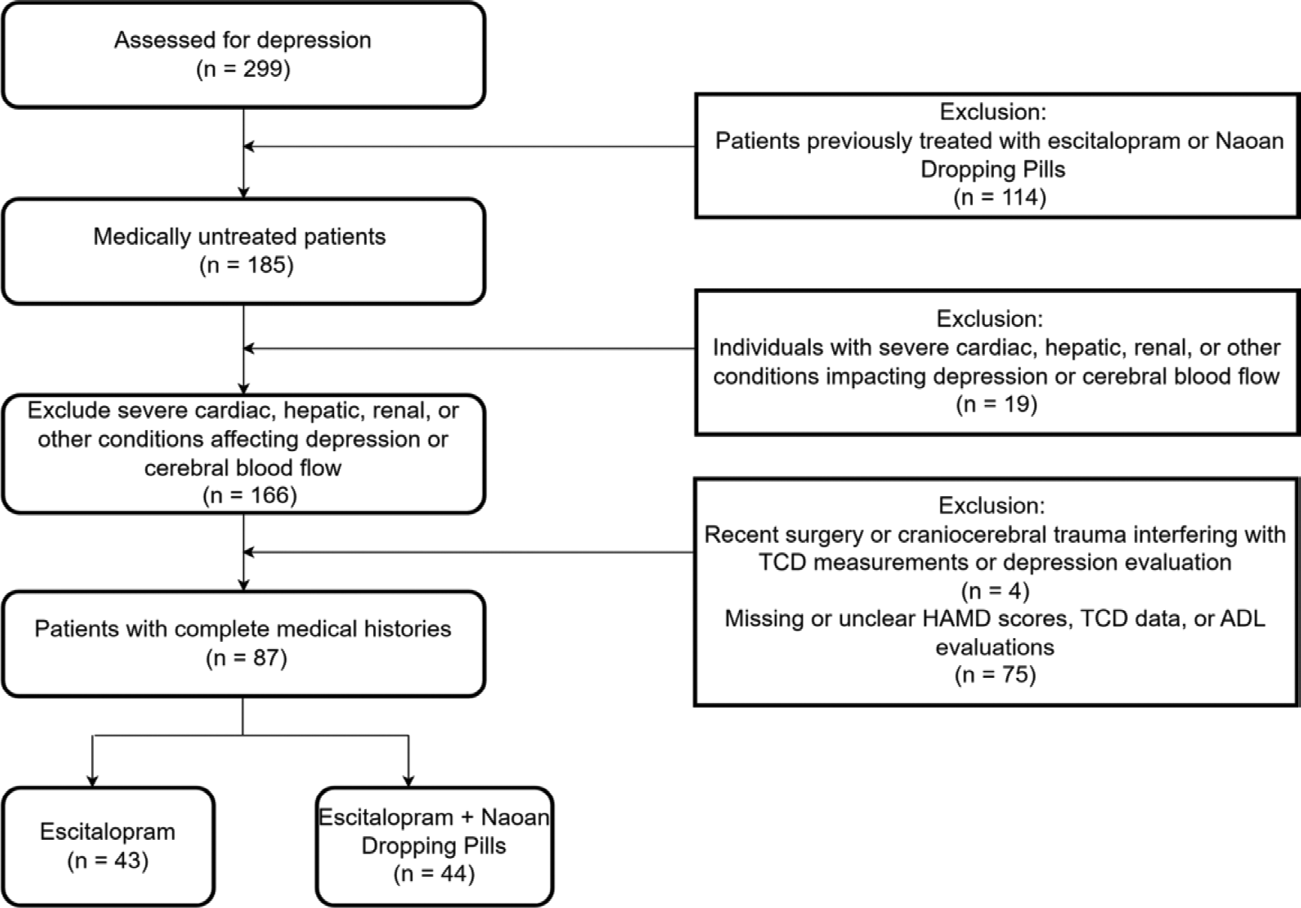
A flowchart of our retrospective study design.

This study was a retrospective analysis of the clinical records of patients diagnosed with depression at The Third People’s Hospital of Tianshui between September 2021 and September 2023. A total of 87 patients were included and divided into 2 treatment groups: Group A (n = 43) and Group B (n = 44) (Fig. 1). Patients in Group A treated with escitalopram (approval number: H20080788), a SSRI. All medications were sourced from Sichuan Kelun Pharmaceutical Co., Ltd. Initially, patients in Group A received a therapeutic dose of 10 mg/day. This dosage was strategically increased to 20 mg/day after 1 week, a common practice, to mitigate initial side effects and gradually adapt the body to medication. The entire course of treatment spanned 2 months, a duration considered adequate to observe the full therapeutic effects of the medication.

In addition to escitalopram (following the same protocol as Group A), patients in Group B received Naoan Dropping Pills, a traditional Chinese medicinal formulation consisting of multiple herbal components (Chuanxiong, Safflower, Angelica, Ginseng, and Borneol). Naoan drop pills (approval number: Z20030121) were purchased from Liaoyuan Yulong Yadong Pharmaceutical Co., Ltd.. The Nanoan dropping pills were administered orally, 2 times a day, with each dose consisting of 20 pills. The duration of this combined treatment was also set at 2 months, aligned with the treatment period of Group A.

### 2.2. Inclusion/exclusion criteria

This retrospective study followed a set of clearly defined inclusion and exclusion criteria, ensuring the scientific rigor and relevance of its findings. The inclusion criteria were as follows: all patients met the diagnostic criteria for depression according to the International Classification of Diseases and Diagnostic Criteria (ICD-10),^[12,13]^ ensuring a uniform study population; and The Hamilton depression scale (HAMD) score was >7.^[14]^ The age range of the participants was set between 35 and 55 years, a decision made irrespective of sex, with the aim of focusing on a demographic that is often significantly impacted by depression; clear documentation indicating the use of escitalopram alone (Group A) or escitalopram plus Naoan dropping pills (Group B) for at least 2 months; and availability of baseline and posttreatment HAMD scores, TCD assessments, and activities of daily living (ADL) scale evaluations, ensuring a comprehensive dataset for each patient.

The exclusion criteria were designed to maintain the integrity of the study by eliminating potential confounding variables. These criteria included patients who had already received escitalopram or Naoan Dropping Pills before the study period to prevent confounding from preexisting treatment effects; patients with severe cardiac, hepatic, renal, or other conditions that might independently affect depression or cerebral blood flow; recent surgical history or any craniocerebral trauma that could interfere with TCD measurements or depression evaluation; and missing or ambiguous HAMD scores, TCD data, or ADL evaluations that would make outcome assessment unreliable.

### 2.3. Observation indicators

#### 2.3.1. Evaluation of efficacy

In this study, we meticulously evaluated the effectiveness of various treatment strategies for depression. Data from scales recorded in electronic medical records prior to the initiation of treatment and 2 months posttreatment were analyzed to comprehensively assess the impact of treatment on each patient.

The primary tools for this evaluation were the HAMD for assessing the severity of depressive symptoms and cerebral blood perfusion parameters to gauge the physiological changes associated with treatment. The HAMD score is a widely recognized measure in clinical psychiatry that offers a detailed assessment of depression severity by quantifying symptoms such as mood, feelings of guilt, suicidal ideation, insomnia, agitation or retardation, anxiety levels, and weight loss.^[15]^ Cerebral blood perfusion parameters were included to understand the effect of treatment on brain physiology, an innovative approach considering emerging evidence linking depression with altered cerebral hemodynamics.

The criteria for determining treatment effectiveness were clearly defined. Effective: A patient’s condition was considered effectively treated if there was a significant improvement in depressive symptoms, demonstrated by a decrease of ≥ 50% in the HAMD score from the baseline.^[16]^ Additionally, an improvement in hemodynamic indices was required, indicating not only a psychological response, but also a physiological response to treatment. Ineffective: The treatment was deemed ineffective in patients who did not exhibit a significant improvement in their depressive symptoms. This was quantified as <50% reduction in the HAMD score compared to baseline, along with stagnant or unimproved hemodynamic indices. The “effective rate” was calculated using the formula: number of effective cases/ total number of included cases × 100%. This metric provides a straightforward, quantitative measure of the treatment success rate, enabling researchers to draw meaningful conclusions about the efficacy of escitalopram alone and in combination with Naoan dropping pills.

#### 2.3.2. Assessment of daily living ability

The daily living ability score scale (ADL) was employed as a key tool in this study to assess a broad spectrum of functionalities among patients with depression, spanning physical and mental health states, mental state, daily communication ability, and daily hands-on ability.^[17]^ The ADL scale was designed with a total score of 100 points, with a higher score signifying greater ability in daily living activities. This scoring system provides a quantifiable measure of a patient’s independence and capacity to perform everyday tasks, which is particularly relevant in the context of depression treatment, where improvements in daily functioning are a significant indicator of recovery. In this study, ADL scores were recorded at 2 critical juncturesafter the first and second months following the initiation of drug intervention. This timing was chosen to monitor progression and any changes in the patients’ abilities to manage daily life activities over the course of treatment. By comparing these scores before and after treatment, researchers aimed to capture the direct impact of the medication (escitalopram alone and in combination with Naoan dropping pills) on the daily living capabilities of the patients.

#### 2.3.3. Hemodynamic evaluation

Transcranial Doppler ultrasound measurements were integrated into the standard clinical practice at The Third People’s Hospital of Tianshui, where they are routinely performed before or during antidepressant therapy for patients diagnosed with depression. TCD scans were performed by 2 board-certified ultrasound technologists, each with accredited diagnostic medical sonography training, national certification, and >5 years of cerebrovascular ultrasound experience. A SIEMENS-SEQUOIA 512 color Doppler ultrasound system (5 to 10 MHz probe, 1.5 mm sampling volume) was used and regularly calibrated according to the manufacturer ’sguidelines. Patients were positioned supine with the head rotated contralaterally, and the bilateral middle cerebral, anterior cerebral, and posterior cerebral arteries were examined following a standardized TCD protocol (consistent insonation angles and standardized depth settings). Hemodynamic evaluations were conducted before and after treatment.

### 2.4. Ethical statement

This study was a retrospective analysis of patient data obtained from electronic medical records at The Third People’s Hospital of Tianshui. All patient information was de-identified before analysis, and no additional data were collected specifically for research purposes. The Institutional Review Board of the Third People’s Hospital of Tianshui waived the requirement for informed consent because of the retrospective design and use of anonymized data. All procedures followed applicable guidelines and regulations, including the Declaration of Helsinki. As part of our routine clinical protocol, patients were informed that their anonymized data could be used for research and quality improvement. Throughout the study, patient confidentiality was strictly maintained and access to individual records was restricted to authorized personnel.

### 2.5. Statistical methods

All data were analyzed using SPSS version 23.0 software (IBM Corp., Armonk). Categorical data are expressed as percentages (%) and analyzed using the chi-square test. For normally distributed continuous variables, independent samples *t*-tests were conducted to compare the means between Group A (escitalopram alone) and Group B (escitalopram + Naoan dropping pills). Statistical significance was set at *P* < .05. significant.

## 3. Results

### 3.1. Baseline characteristics of participants

The mean age of the A group was 44.49 ± 5.42 years old and the mean age of the B group was 45.64 ± 6.84 years old. The course of disease was 4.46 ± 1.96 years in A group, and 5.14 ± 2.34 years in group B. There were no statistical differences between the 2 groups in terms of age, gender, education duration, BMI, and disease course, between the group A and the group B (*P* > .05). Further details are listed in Table 1.

**Table 1:** Comparison of general information between the 2 groups.

Characteristics	A (n = 43)	B (n = 44)	*P*-value
Age (yr, x ± s)	44.49 ± 5.42	45.64 ± 6.84	.389
Sex (n)			
Female	22 (51.16%)	22 (50.00%)	.914
Male	21 (48.84%)	22 (50.00%)	
Education duration (yr)	5.48 ± 3.47	6.14 ± 3.12	.394
BMI (kg/m^2^)	21.40 ± 1.60	22.50 ± 1.59	.097
Disease course (yr, x ± s)	4.46 ± 1.96	5.14 ± 2.34	.238

Data are expressed as mean ± standard deviation. Sex was compared using the chi-square test, and other parameters were compared using Student *t* test. *P < .05.

BMI = body mass index.

### 3.2. Comparison of efficacy between the 2 groups

This retrospective analysis showed that, based on HAMD scores recorded after treatment, the effective rate in Group B was 90.91% (40/44), which was higher than that of Group A at 72.09% (31/43). The difference in treatment efficacy between the 2 groups was statistically significant (χ² = 3.953, *P* = .047). These findings suggest that escitalopram plus Naoan Dropping Pills may have a more significant effect on improving depressive symptoms than escitalopram alone. Further details are presented in Table 2. details.

**Table 2:** Comparison of curative effects after intervention between the 2 groups.

Group	Effective (n)	Ineffective (n)	Effective rate (%)
A group (n* *= 43)	31 (72.09)	12 (27.91)	72.09
B group (n* *= 44)	40 (90.91)	4 (9.09)	90.91
*χ* ^*2*^			3.953
*P*			.047

### 3.3. Comparison of hemodynamics between the 2 groups

Before the intervention, the mean systolic blood flow velocities in the major cerebral arteries (MCA), ACA, and PCA in Group A were not significantly different from those in Group B. After the intervention, the MCA, ACA, and PCA systolic mean blood flow velocities in Group A were significantly lower than those in Group B. Further details are presented in Table 3. details.

**Table 3:** Comparison of systolic blood flow velocity between the 2 groups after intervention (x ± s).

Group	A group (n = 43)		B group (n = 44)		*P*	
	Left	Right	Left	Right	Left	Right
MCA						
Before intervention	92.64 ± 9.56	92.34 ± 9.15	90.74 ± 9.47	91.03 ± 9.41	.3578	.5149
After intervention	94.38 ± 7.01	94.78 ± 5.47	98.27 ± 5.65	97.96 ± 6.16	.0062	.0131
ACA						
Before intervention	69.64 ± 7.39	70.85 ± 4.82	70.24 ± 5.40	70.69 ± 4.71	.6684	.8799
After intervention	72.49 ± 6.01	73.88 ± 4.85	75.31 ± 4.31	78.65 ± 4.18	.0155	<.0001
PCA						
Before intervention	59.25 ± 5.99	59.50 ± 6.90	58.44 ± 6.04	58.67 ± 6.80	.5320	.4050
After intervention	65.74 ± 4.81	61.32 ± 4.45	68.43 ± 3.82	63.94 ± 3.92	.0057	.0067

ACA = anterior cerebral artery, MCA = middle cerebral artery, PCA = posterior cerebral artery.

* *P* < .05.

Before the intervention, there was no significant difference in the MCA, ACA, and PCA diastolic blood flow velocities between groups A and B. After the intervention, the mean diastolic blood flow velocity of the MCA, ACA, and PCA in group A was significantly lower than that in group B. Further details are presented in Table 4. details.

**Table 4:** Comparison of diastolic blood flow velocity between the 2 groups after intervention (x ± s).

Group	A group (n = 43)		B group (n = 44)		*P*	
	Left	Right	Left	Right	Left	Right
MCA						
Before intervention	38.86 ± 4.14	39.38 ± 5.44	38.05 ± 4.37	38.04 ± 4.18	.4031	.2262
After intervention	40.57 ± 4.08	40.78 ± 4.43	42.63 ± 3.94	43.89 ± 4.74	.0209	.0026
ACA						
Before intervention	30.06 ± 3.58	29.29 ± 5.62	29.44 ± 4.10	29.59 ± 5.85	.4845	.8168
After intervention	31.17 ± 3.44	31.29 ± 4.57	33.67 ± 2.96	34.31 ± 3.85	.0006	.0015
PCA						
Before intervention	25.14 ± 2.70	25.16 ± 4.23	24.39 ± 2.60	23.83 ± 4.08	.2138	.1644
After intervention	27.37 ± 2.93	26.43 ± 2.74	29.11 ± 2.23	28.40 ± 3.20	.0030	.0033

ACA = anterior cerebral artery, MCA = middle cerebral artery, PCA = posterior cerebral artery.

* *P* < .05.

**Figure 2.**
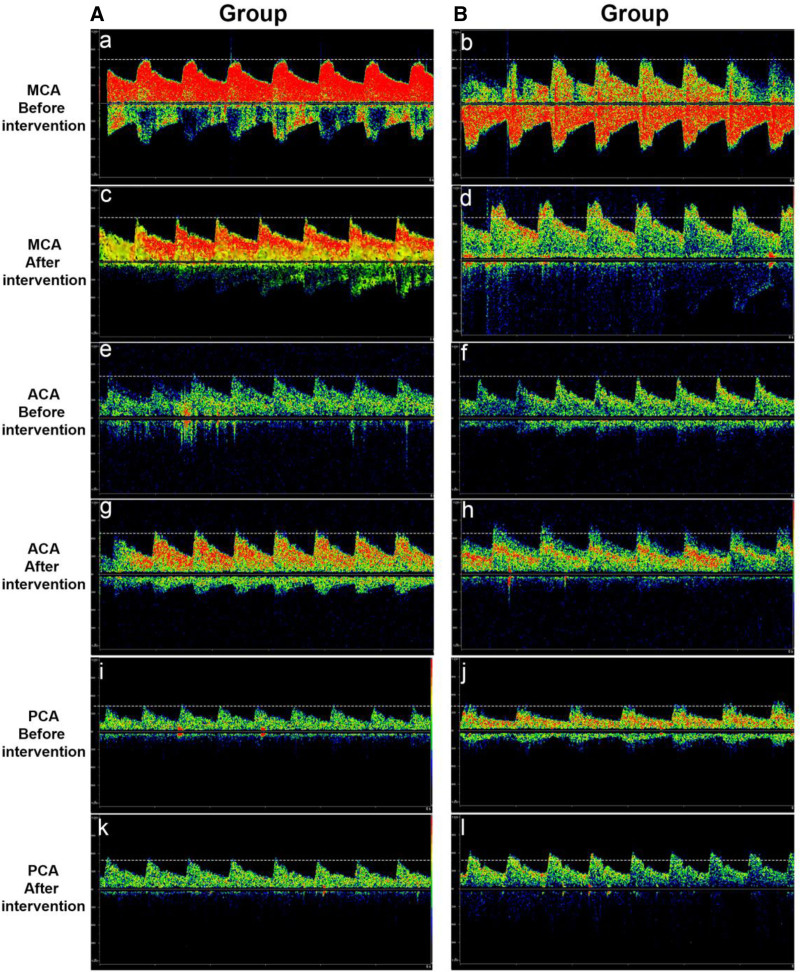
Vascular ultrasound spectrogram: Group A is shown on the left, and Group B on the right. (A and B) Ultrasound images of the middle cerebral artery before treatment; (C and D) posttreatment ultrasound images of the middle cerebral artery; (E and F) Ultrasound images of the anterior cerebral artery before treatment; (G and H) posttreatment ultrasound images of the anterior cerebral artery; (I and J) Ultrasound images of the posterior cerebral artery before treatment; (K and L) Ultrasound images of the posterior cerebral artery after treatment.

Spectral images before and after the intervention are shown in Figure 2A-l. Before the intervention, the mean blood flow velocity in the systolic and stretch phases of the MCA of patients in groups A and B were at the same baseline level (Figs. 2A and B); after the intervention, the systolic and diastolic blood flow velocities in group B were higher than those in group A, and the spectral peak value in group B was higher than that in group A (Figs. 2C and D). The same phenomenon was observed for ACA and PCA. Before the intervention, the systolic and diastolic blood flow velocities of the ACA and PCA in groups A and B were at the same baseline level, while after the intervention, the systolic blood flow velocity and diastolic blood flow velocity of group B were higher than those of group A, which was manifested in a higher spectral peak value of group B than that of group A (Figs. 2E-l).

### 3.4. Comparison of daily living ability between the 2 groups

By comparing the ADL scores, we found no significant difference between groups A and B before the intervention. One and 2 months after the intervention, the level of daily living ability of patients in group B was higher than that in group A, especially 2 months after the intervention, and the level of daily living ability of patients in group B was more significant than that in group A (see Table 5 for details).

**Table 5:** Comparison of daily living ability before and after intervention between the 2 groups (x ± s).

Group	A group (n = 43)	B group (n = 44)	*P*
ADL score			
Before intervention	57.07 ± 4.95	57.11 ± 4.77	.9655
1 mo after intervention	66.59 ± 5.51	71.32 ± 5.71	.0002
2 mo after intervention	73.11 ± 5.56	79.48 ± 5.60	<.0001

ADL = ability of daily living.

* *P* < .05.

## 4. Discussion

This study retrospectively investigated the effects of escitalopram combined with Naoan dropping pills on depressive symptoms and cerebral hemodynamics in 87 patients with depression. The results showed that the efficacy rate in the combination treatment group was significantly higher than that in the escitalopram single treatment group, and the levels of daily living ability and cerebral hemodynamic indices were also improved. These findings highlight the importance of a comprehensive treatment approach for depression.

As an SNRIs drug, escitalopram has high selectivity, quick effect, and safety, and can improve patients’ depression in a short time.^[18,19]^ As a proprietary drug, the Naoan dropping pill accelerates cerebral blood flow, protects vascular endothelia, supplements qi, and clears collages. It can effectively slow brain injury caused by ischemia.^[20]^ The Naoan dropping pill is a specialized formulation of TCM crafted from a quintet of potent herbal components, each selected for its distinctive therapeutic properties. This blend includes chuanxiong (Ligusticum wallichii), safflower (Carthamus tinctorius), angelica (Angelica sinensis), ginseng (Panax ginseng), and Borneol.^[20]^ Chuanxiong is known for its efficacy in promoting blood circulation and facilitating the smooth flow of qi and blood through the meridians of the body.^[21]^ Safflower is utilized for its potent blood-stasis-removal properties. It works synergistically with chuanxiong to enhance blood flow.^[22]^ Borneol is known to have beneficial properties. It is believed to open the orifices, clear the mind, and facilitate the deeper penetration of other herbs into body tissues.^[23]^ In this study, it was found that the total effective rate of 90.91% (40/44) in group B was more significant than that of 72.09% (31/43) in group A after this treatment intervention, and the curative effect of the 2 groups was significant (χ^2^ = 3.953, *P* = .047). One and 2 months after the intervention, the daily living ability of group B patients was higher than that of group A patients; in particular, the daily living ability of group B patients was significantly better than that of group A patients (*P* < .0001). All the above indicate that the efficacy and daily living ability of the patients after applying the combined intervention program were superior to those of the single-drug intervention program.

Some researchers have indicated that CBFSR is impaired in patients with depression, and many neurological symptoms in patients with depression are related to impaired CBFSR.^[1]^ Changes in the cerebral blood flow play an important role in the occurrence and development of depression. Studies have shown that certain brain regions in patients with depression, especially those related to emotional regulation (e.g., the prefrontal lobe, hippocampus, and amygdala), tend to show reduced cerebral blood flow. This decrease in cerebral blood flow may be associated with depressive symptoms, such as emotional regulation disorders and cognitive function decline.^[24]^ Therefore, changes in cerebral blood flow are not only a result of depression, but may also be 1 of the factors that promote the development of depression. Regarding whether improved cerebral blood flow has positive implications for the treatment of patients with depression, existing research suggests that improved cerebral blood flow may have a positive impact. Some treatments, such as cognitive behavioral therapy, medication, and physical therapy (such as transcranial magnetic stimulation, transcranial direct current stimulation, etc), have been found to reduce depressive symptoms by improving cerebral blood flow.^[25,26]^ This finding suggests that interventions targeting the cerebral blood flow may be effective in treating depression. Unlike other neuroimaging techniques (e.g., fMRI, PET, or SPECT), TCD is portable, relatively inexpensive, and entirely noninvasive, making it a practical choice for repeated measurements.^[27]^ This allows for ongoing monitoring of hemodynamic changes over the course of treatment. TCD directly measures the velocity of blood flow in the MCA (ACA and PCA) in real-time. Changes in these velocity readings can be interpreted as indicators of altered resistance or compliance in the cerebral vasculature, which in turn may correlate with shifts in a patient’s clinical status. Psychiatric outcomes often rely on self-report or interviewer-administered symptom scales (e.g., HAMD). Although these are indispensable, objective TCD hemodynamic parameters can provide physiological corroboration. Improvements in blood flow parameters may support the clinical gains observed on the subjective scales. Therefore, the use of TCD ultrasonography to detect cerebral hemodynamic changes in patients is of great significance for evaluating their condition. In this study, after the intervention, the systolic blood flow velocity in group B was significantly higher than that in group A. Following the intervention, the diastolic blood flow velocity in group B was significantly higher than that in group A. This indicates that after the combined intervention treatment, the patients’ systolic and diastolic blood flow velocities were significantly increased by TCD ultrasound detection and their depressive symptoms improved.

Although this study provides valuable insights into the effects of escitalopram combined with Naoan dropping pills on cerebral hemodynamics in patients with depression, it has some limitations. We limited enrollment to individuals aged 35 to 55 years because depression commonly manifests in midlife and minimized confounding factors associated with younger or older cohorts. This restriction helped reduce comorbid conditions (e.g., cardiovascular or neurodegenerative disorders) that could affect treatment outcomes and Transcranial Doppler measurements. It also ensured more consistent assessments of ADL, as baseline activity levels can differ substantially between younger and older groups. However, our narrow age range may limit the generalizability to other populations, potentially underestimating or overestimating the treatment effects in younger or older patients. We acknowledge that this reduced diversity could have affected the broader applicability of our findings. Only 87 patients were included in this study, limiting the general applicability and statistical power of the results. Larger sample sizes may provide more robust data analyses and more representative results. Studies have focused mainly on short-term treatment outcomes and lack data on the long-term follow-up of patients. Long-term follow-ups can provide important information regarding treatment continuity and safety. The absence of a placebo control group may have affected the evaluation of the effectiveness of the study. A placebo control group can help determine the relationship between the treatment effect and the patient’s expected effect. In addition, studies mainly rely on TCD ultrasound detection and scale scores as evaluation indicators and may lack the evaluation of other objective biomarkers, such as neurotransmitter levels and inflammatory markers, which can provide more biological evidence for treatment effects. In addition, future studies could stratify patients according to factors such as age, depression severity, or comorbid conditions to determine which subgroups derive the greatest benefit from combined treatment protocols.

## 5. Conclusion

Combining escitalopram with Naoan dropping pills significantly improved depressive symptoms, daily functioning, and cerebral hemodynamics compared with escitalopram alone. Although the small sample size, narrow age range, and lack of a placebo control group limit the generalizability of these findings, they underscore the potential of integrating TCM with standard antidepressant therapy. Larger, placebo-controlled, and long-term studies are recommended to validate and extend these results.

## Author contributions

**Conceptualization:** Xiaoyan Wang.

**Data curation:** Yan Che, Wenli Wang.

**Formal analysis:** Yan Che.

**Funding acquisition:** Xiaoyan Wang.

**Investigation:** Xiaoyan Wang.

**Methodology:** Xiaoyan Wang, Yan Che, Wenli Wang.

**Project administration:** Xiaoyan Wang.

**Resources:** Xiaoyan Wang.

**Software:** Xiaoyan Wang.

**Supervision:** Xiaoyan Wang.

**Writing – original draft:** Xiaoyan Wang.

**Writing – review & editing:** Xiaoyan Wang, Yan Che, Wenli Wang.
